# Synergistic Effects of International Oil Price Fluctuations and Carbon Tax Policies on the Energy–Economy–Environment System in China

**DOI:** 10.3390/ijerph192114177

**Published:** 2022-10-30

**Authors:** Shu Mo, Ting Wang

**Affiliations:** 1 School of Management, Guizhou University, Guiyang 550025, China; 2 Guizhou Provincial Key Laboratory of Internet Plus Intelligent Manufacturing, School of Mechanical Engineering, Guizhou University, Guiyang 550025, China

**Keywords:** international oil price, carbon tax policy, energy–economy–environment, CGE model, synergistic effects

## Abstract

Catalyzed by COVID-19 and the Russia–Ukraine conflict, oil prices fluctuate dramatically on the worldwide market. Both international oil price changes and carbon tax policies have a direct impact on energy costs, thus influencing energy security and emission reduction impacts. Therefore, assessing the interaction effects of international oil price variations and carbon tax policies can assist in resolving the competing challenges of energy security and carbon emission reduction. The impact of international oil price fluctuations on China’s energy–economic–environment system under the baseline scenario and carbon taxation scenario is analyzed by constructing a computable general equilibrium model comprising six modules: production, trade, institutions, price, environment, and equilibrium. The findings indicate that, in addition to reducing high-carbon energy consumption and increasing demand for clean electricity, rising international oil prices have a negative effect on real GDP, resulting in lower output in sectors other than construction, and a positive effect on the environmental system by driving carbon emission reductions. In contrast, decreasing international oil prices have the opposite effect. Nevertheless, the impact of rising and decreasing international oil prices is asymmetrical, with the positive shock effect being smaller than the negative. The carbon tax policy can effectively offset the increase in carbon emissions caused by the decline in international oil prices, which is conducive to promoting the development of clean energy, while simultaneously causing an increase in product prices and arousing a contraction in consumer demand, which has a limited negative impact on the macroeconomy.

## 1. Introduction

**Figure 1:**
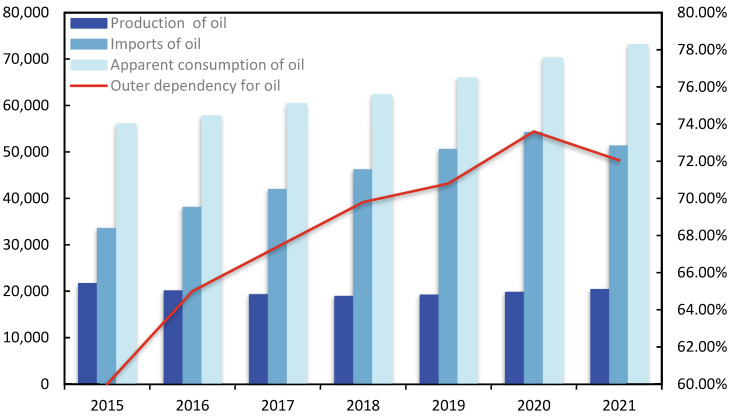
The supply and demand situation for oil in China.

Oil is a critical resource that ensures a country’s economic and social growth and plays a vital role in production and survival. Oil security is intimately tied to a country’s future and fate [1]. As people’s living standards continue to rise and industrialization advances, China, the world’s top crude oil importer, has a widening disparity between supply and demand for crude oil. As indicated in Figure 1, China’s crude oil import dependency was 72.05% in 2021, which is much higher than the International Energy Agency’s warning line for crude oil import dependency [2], thus international oil market fluctuations will have a stronger influence on China’s oil security.

**Figure 2:**
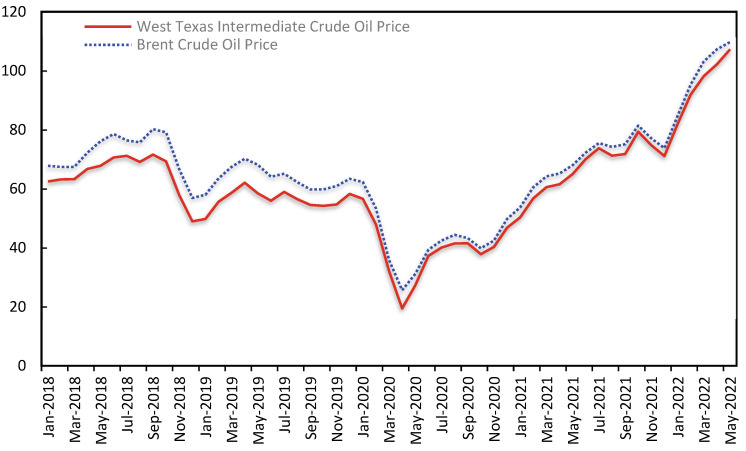
Trend in International Oil Prices.

The international volatility of oil prices is the direct source of oil security issues [3]. China’s oil imports are too reliant on the Middle East, Russia, and other nations, as well as the majority of oil import channels being located in areas that are particularly susceptible to international political disputes. As seen in Figure 2, international crude oil prices fell fast in October 2018 and reached a new low in December, mostly owing to the diminished influence of the U.S. crude oil embargo on Iran on the international crude oil market and the continuous growth of U.S. crude oil stockpiles. Early in the year 2020, a global outbreak of Newcastle pneumonia disrupted global economic activity, depressed crude oil demand in April, and precipitated a precipitous decline in international oil prices. Early in 2022, the commencement of the Russia–Ukraine conflict as well as the application of multiple sanctions on Russia by the U.S. and several European nations led to a jump in international oil prices, which at one time reached USD 120 per barrel, a new high since 2008. The vast and dramatic variations in oil prices indicate that traditional oil geopolitical competition and games continue to exist, that the international market situation is complicated and unpredictable, and that China’s oil supply continues to be vulnerable to a number of unstable causes [4]. Consequently, it is of enormous practical importance to examine the effects of international oil price volatility on different sectors of the national economy.

On the other hand, the ongoing discussion over global climate change will influence oil demand forecasts. If the world continues on its present trajectory of carbon emission intensity and greenhouse gas rises, one-third of the global population might experience excessive heat by 2070, according to Xu et al. [5]. In this context, the Chinese government has proposed a carbon peak target for 2030, a carbon neutral vision for 2060, and new initiatives to increase the country’s autonomous contribution, which not only demonstrates China’s great power role in combating global climate change but also demonstrates the severe test of China’s development posed by international public opinion pressure and green trade barriers in the external environment [6]. Attaining carbon peak and carbon neutral objectives is a long-term, slow, and difficult process, and aggressive emission reduction strategies may create classic oil security shocks, such as increasing oil prices [7]. Although oil makes for a relatively small fraction of China’s primary energy consumption now, overall oil consumption is significant and the amount of oil imports is substantial. With the further optimization of China’s economy and energy structure, oil demand will stay robust for a period of time in the future, and thus the proportion of oil used will climb substantially [8]. In the process of a rapid low-carbon energy transition, China will unavoidably face short- or medium-term oil security threats. To combine the concerns of oil security assurance with low-carbon growth is a desirable endeavor.

Experience has demonstrated that a variety of climate policies, such as GHG emission reduction targets and emission regulations, can serve as essential incentives to combat climate change but can be counterproductive if the implementation process is flawed [9]. Carbon taxes and carbon trading are the primary economic measures implemented internationally to decrease carbon emissions [10,11]. The national carbon emission trading market was formally inaugurated on July 16, 2021, marking a new phase in China’s carbon emission administration. The carbon tax is an environmental tax placed on the quantity of carbon in fossil fuels or carbon dioxide emissions; however, China has not yet implemented carbon tax policies into national carbon emission reduction initiatives. Although China’s carbon market is projected to be expanded in the future to cover petrochemicals, chemicals, building materials, aviation, and other vital industries, sources accounting for 50% of the country’s total carbon emissions will not be included [12]. In this context, carbon tax, as an additional essential measure of emission reduction, has a significant role to play in China’s future carbon emission control system [13,14]. The carbon tax policy will have an instant influence on the price of oil, which will have repercussions for energy security and emission reduction. In light of international oil price variations, it is of major practical importance to examine the effect of carbon tax policy on different sectors of the national economy.

However, current research tends to concentrate on the effects of oil price fluctuations and carbon tax policies on energy consumption, economic factors, and environmental factors, respectively, under the assumption of constant external conditions [15,16]. That is, they do not examine the interaction between the two when measuring oil price fluctuations or evaluating carbon tax policies. In light of this, the following features represent the paper’s marginal contributions. First, a computable general equilibrium model is built using the recently published Chinese input–output table, as well as an environmental module being added to the model to investigate carbon emissions. This approach outperforms previous research in terms of how effectively it captures the environmental effects of changes in international oil prices and carbon tax policies during the synergistic process. Second, this paper is the first to use a computable general equilibrium model to simulate the effects of carbon tax policies and fluctuations in the price of oil on China’s energy, economic, and environmental systems, as well as their interactions. This helps to resolve the competing concerns of oil security and carbon emission reduction. Third, the conclusions of this study offer a scientific foundation and a decision-making guide for the timing of China’s future carbon tax policy, in addition to completing the body of current knowledge.

This research is organized as follows. Section 2 is the literature review. Section 3 illustrates the model construction and data sources. The scenario setting and simulation results of the analysis are illustrated in Section 4. Finally, Section 5 concludes with recommendations.

## 2. Literature Review

International oil price fluctuation has been the focus of a sizable number of studies to date and these studies’ breadth and research perspectives have been growing, mostly in relation to the following issues.

The first category investigates how changes in the price of oil on the global market affect energy security, energy structure optimization, and energy consumption. The short-term unidirectional association between oil prices and the use of renewable energy was validated by Brini et al. [17]. Energy efficiency, oil pricing, and environmental concerns all have different effects on renewable energy, according to research by Wang et al. [18]. Thorbecke explored the mechanisms by which oil price volatility affects the energy security of Asian economies to help them withstand the impact of oil price changes [19]. Mensah et al. discovered bilateral causal correlations between oil prices and economic growth, energy use, and carbon emissions over the long and short term [20]. According to McCollum et al., persistently high or low oil prices might have a major impact on the global energy system in the ensuing decades [21].

The second category focuses on the effects of fluctuations in the price of oil on the macroeconomy and different industrial sectors. Significant nonlinear impacts of international oil prices on Chinese economic development and inflation were discovered by Du et al. [22]. More than a century’s worth of data from industrialized nations was used by Van et al. to examine the strong and weak correlation between oil price changes and economic growth [23]. The direction, scope, and duration of the macroeconomic impact of oil price volatility were investigated by Mohaddes et al. [24]. The industrial, energy, gas, and water industries in Colombia are more severely impacted than other sectors, according to Otero’s research [25]. The causal link between systemic risk in the crude oil market and uncertainty in global economic policy was proven by Yang et al. [26].

The third category focuses on examining how fluctuations in the price of oil on a global scale affect environmental problems such as air pollution. In ten nations with large carbon emissions, including China, the United States, and Canada, Ullah et al. carefully examined the asymmetric impacts of oil price variations on environmental pollution [27]. International oil prices and carbon emissions are inversely causally connected, according to Malik et al. [28].

Research on carbon taxes has increasingly expanded from a solely environmental perspective to various aspects, such as energy and economy, with a primary focus on the following.

The first category looks at how carbon tax impacts several aspects of energy use, including energy mix and production efficiency. Di et al. investigated how two distinct carbon tax scenarios might affect the Irish energy industry [29]. The time of the carbon tax’s adoption is crucial, as demonstrated by Fang et al. The better the timing of the tax’s implementation, the simpler it will be to regulate carbon emissions and the more energy intensity will decline [30]. To examine the effects of the carbon tax on China’s energy, environment, and economy, Lin et al. created a number of scenarios for various carbon tax rates and taxable industries [31]. According to Matsumoto’s argument, a carbon tax would have an impact on families’ energy consumption decisions by altering the relative costs of different energy sources [32]. A carbon tax would have a higher influence on electricity use than on the use of fossil fuels, according to research by Zhang et al. on how it might affect China’s tourism-related energy consumption [33].

The second category evaluates the macroeconomic effects of the carbon price as well as its effects on small businesses. The effects of the carbon price on the Chinese economy and the protective effect of supplementary measures were studied by Lu et al. [34]. In order to determine how a carbon tax might affect the Chilean economy, Benavente carried out a cross-sectional analysis [35]. The correlation between carbon tax, GHG emission reduction, and economic development was quantitatively analyzed by Liu et al. after simulating various carbon tax rates [36]. A moderate carbon tax policy would have a large influence on emissions and a minor impact on the economy, according to Wei’s investigation of the effects of a carbon tax, a climate change policy, on the Chinese economy in the post-epidemic era [37].

The third category is devoted to research on carbon tax policies’ ability to reduce carbon emissions and thus enhance environmental quality. Carbon taxes considerably slowed the development of per capita CO_2_ emissions in Finland, according to Lin et al. [38]. A stepped carbon price is more effective in cutting pollution, according to Zhai’s analysis of the connections between carbon tax, energy, and climate variables [39]. However, Nie et al. discovered that carbon prices may not always help the environment and might perhaps do harm [40].

However, previous research on the effects of oil prices on the energy, economic, and environmental systems can help China strengthen its ability to withstand fluctuations in oil prices in a complex environment for economic policy and offer useful theoretical support for the steady growth of China’s economy. For the purposes of reaching carbon peaking and carbon neutrality as well as for comprehending its function in climate governance, historical research on the effects of carbon tax policy on energy, economic, and environmental systems is crucial. It is not difficult to discover, nevertheless, that there is no research literature on the combined synergistic effects of international oil prices and carbon tax policies on energy, the economy, and the environment. The hunt for the ideal timing to implement carbon tax policies necessitates ongoing attention and analysis, especially now that changes in the price of oil internationally have become commonplace. Therefore, this paper is an addition to the relevant research on the effects of carbon tax policies on different sectors of the national economy in the context of the volatility of the international oil price. This paper will build a computable general equilibrium model taking into account six modules: production, trade, institutions, price, environment, and equilibrium. It will then systematically analyze the synergistic impact mechanism of the international oil price fluctuation and carbon tax policy on China’s energy–economy–environment system.

## 3. Model Construction and Data Description

### 3.1. CGE Model

Computerized general equilibrium models are able to integrate the analysis of various national economic sectors and economic cycles and investigate the mechanisms that drive and restrain one another. Therefore, to study the synergistic influence mechanism of the international oil price fluctuation and carbon tax policy on China’s energy–economy–environment system, a computable general equilibrium model will be built, taking into account six modules: production, trade, institutions, price, environment, and equilibrium.

**Figure 3:**
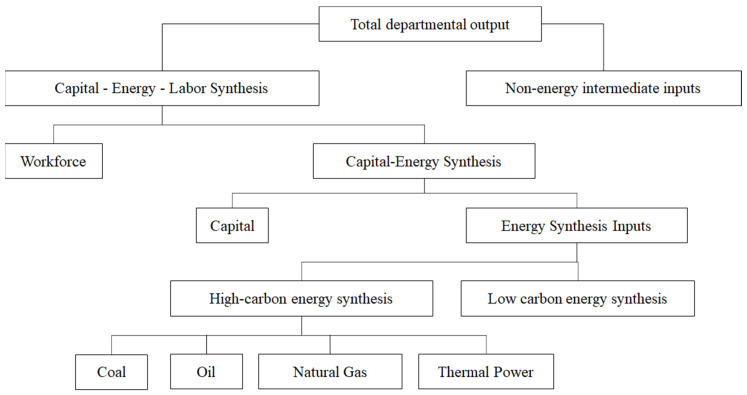
Schematic diagram of the structure of the production function.

As seen in Figure 3, the production module is divided into five layers of nested functions: the first layer synthesizes capital–energy–labor and non-energy intermediate inputs; the second layer synthesizes labor inputs and capital–energy; the third layer synthesizes capital and energy; the fourth layer synthesizes high-carbon energy and low-carbon energy; the fifth layer synthesizes coal, oil, natural gas, and thermal power. Each layer is in the form of a CES function.

The production function used for the synthesis of the inputs of each layer is as follows.

(1) $$\begin{array}{c} QX_{i}={\left[\beta_{i}^{KEL}{\left(\alpha_{i}^{KEL}KEL_{i}\right)}^{\frac{\lambda_{i}^{Q}-1}{\lambda_{i}^{Q}}}+\beta_{i}^{ND}{\left(\alpha_{i}^{ND}ND_{i}\right)}^{\frac{\lambda_{i}^{Q}-1}{\lambda_{i}^{Q}}}\right]}^{-\frac{\lambda_{i}^{Q}}{\lambda_{i}^{Q}-1}} \end{array}$$

(2) $$\begin{array}{c} KEL_{i}={\left[\beta_{i}^{KE}{\left(\alpha_{i}^{KE}KE_{i}\right)}^{\frac{\lambda_{i}^{KEL}-1}{\lambda_{i}^{KEL}}}+\beta_{i}^{L}{\left(\alpha_{i}^{L}L_{i}\right)}^{\frac{\lambda_{i}^{KEL}-1}{\lambda_{i}^{KEL}}}\right]}^{-\frac{\lambda_{i}^{KEL}}{\lambda_{i}^{KEL}-1}} \end{array}$$

(3) $$\begin{array}{c} KE_{i}={\left[\beta_{i}^{K}{\left(\alpha_{i}^{K}K_{i}\right)}^{\frac{\lambda_{i}^{KE}-1}{\lambda_{i}^{KE}}}+\beta_{i}^{E}{\left(\alpha_{i}^{E}E_{i}\right)}^{\frac{\lambda_{i}^{KE}-1}{\lambda_{i}^{KE}}}\right]}^{-\frac{\lambda_{i}^{KE}}{\lambda_{i}^{KE}-1}} \end{array}$$

In the trade module, it is assumed that domestic producers adhere to the maximization of profit principle when determining the optimal quantity of domestic goods supply and exports and that domestic demanders adhere to the maximization of utility principle when determining the optimal quantity of domestic goods demand and imports.

CES production function is used:

(4) $$\begin{array}{c} QX_{i}={\left[\beta_{DA}QD_{i}^{\lambda_{i}^{QE}}+\beta_{EX}QE_{i}^{\lambda_{i}^{QE}}\right]}^{\frac{1}{\lambda_{i}^{QE}}} \end{array}$$

(5) $$\begin{array}{c} QQ_{i}={\left[\beta_{DQ}QD_{i}^{\lambda_{i}^{QM}}+\beta_{IM}QE_{i}^{\lambda_{i}^{QM}}\right]}^{\frac{1}{\lambda_{i}^{QM}}} \end{array}$$

The pricing module has three primary categories of prices: import and export commodity prices, domestic consumer commodity prices, and factor prices.

(6) $$\begin{array}{c} PX_{i}={\left[\beta_{i}^{KEL}PKEL_{i}^{1-\lambda_{i}^{Q}}+\beta_{i}^{ND}PND_{i}^{1-\lambda_{i}^{Q}}\right]}^{\frac{1}{1-\lambda_{i}^{Q}}} \end{array}$$

(7) $$\begin{array}{c} PKEL_{i}={\left[\beta_{i}^{KE}PKE_{i}^{1-\lambda_{i}^{KEL}}+\beta_{i}^{L}PL_{i}^{1-\lambda_{i}^{KEL}}\right]}^{\frac{1}{1-\lambda_{i}^{KEL}}} \end{array}$$

(8) $$\begin{array}{c} PKE_{i}={\left[\beta_{i}^{E}PE_{i}^{1-\lambda_{i}^{KE}}+\beta_{i}^{K}PK_{i}^{1-\lambda_{i}^{KE}}\right]}^{\frac{1}{1-\lambda_{i}^{KE}}} \end{array}$$

The institutional module consists of the income and expenditure functions of residents, firms, and the government, where the consumption function of inhabitants is chosen as the Stone–Gailey utility function and the remaining functions are linear functions with proportional coefficients.

The environmental module evaluates the pollutants emitted during production and consumption by each economic sector. As a calibration in this paper, carbon dioxide is chosen as a common environmental variable. It also relies on Mardani’s analysis of carbon dioxide emissions, which defines carbon emissions as stemming mostly from the final consumption of carbon-containing products.

The equilibrium module consists of labor market equilibrium, capital market equilibrium, and commodity market equilibrium, whereas the closed function module comprises savings–investment equilibrium, government balance of payments, and balance of payments.

### 3.2. Data Description

As computation inputs, the computable general equilibrium model uses the social accounting matrix and related parameters. Using the input–output table of 2020 as the basis of calculation and GTAPAgg software to adjust the input–output table into 11 sectors according to the research needs and industrial characteristics, and with the assistance of relevant data published in the *China Statistical Yearbook* and *China Energy Statistical Yearbook*, this paper constructs a social accounting matrixa necessary for the computable general equilibrium model to examine the effect of international oil price fluctuations and carbon tax on China’s energy–economic–environment system. Table 1 compares the specific sectoral division of the model presented in this research to the sectoral division of the original input–output table. In this paper, the carbon emission factors of fossil energy sources are determined according to the 2006 *IPCC Guidelines for National Greenhouse Gas Inventories.*

## 4. Scenario Formulation and Simulation Evaluation

### 4.1. Scenario Formulation

The synergistic effects of changes in international oil prices, an external economic variable, and carbon tax policies on energy, economy, and environment are examined in this research using scenario analysis. This essay fully considers the significant contemporary influences on the variation in international oil prices. First, the new crown epidemic’s onset has resulted in a sizable number of global company closures, job losses, a reduction in social consumption, and a sizable decline in international oil demand, which has exacerbated the global oil oversupply and caused international oil prices to remain at low levels. The second is the ongoing war between Russia and Ukraine. As a result of sanctions placed on Russia by the U.S. and other Western nations, oil prices have skyrocketed globally.

Additionally, the carbon tax, a price-based approach to carbon pricing, is becoming more and more popular throughout the globe due to its special ability to support governance of the energy transition and climate change. It has also achieved some progress in its implementation. In light of its economic and social development stage and mounting pressure from international climate challenges, China has made carbon peak and carbon neutrality its long-term policy goals. As a result, China needs to introduce carbon tax policy tools to broaden new concepts in carbon governance.

The following policy scenarios are outlined in detail.

Scenario 1: As a result of the Russia–Ukraine conflict’s geopolitical influence, international oil prices continue to grow by 10%, 20%, or 30%.

Scenario 2: The COVID-19 epidemic causes a huge number of worldwide businesses to close, employees to lose their employment, societal consumption levels to decline, international oil demand to collapse, and international oil prices to decrease by 10%, 20%, or 30% of the shock effect.

Scenario 3: Based on Scenario 1, a carbon tax policy is introduced for carbon emissions resulting from the use of fossil energy at a rate of 30 RMB per ton of CO_2_.

Scenario 4: Based on Scenario 2, a 30 RMB/ton CO_2_ carbon tax is placed on carbon emissions induced by the use of fossil fuels.

### 4.2. Impact Analysis of Scenario Combination on Energy

**Figure 4:**
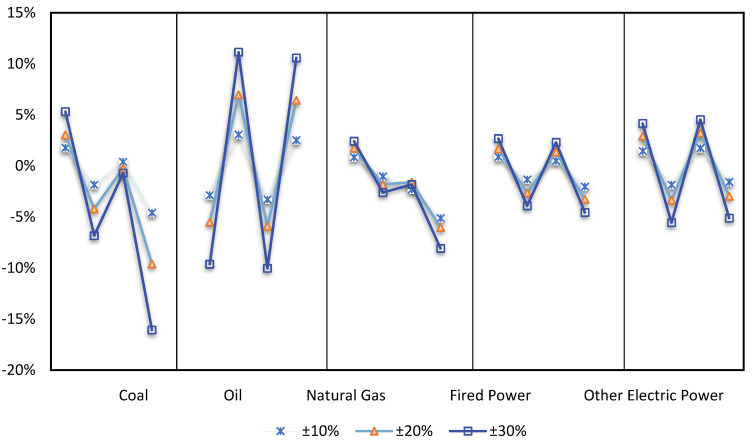
Impact of different scenarios on energy demand and structure Unit: %.

Figure 4 illustrates the various effects that various scenarios have on the amount of energy consumed and the structure. The investigated energy sources are separated into two categories: high-carbon energy sources and low-carbon energy sources. High-carbon energy sources include coal, oil, natural gas, and thermal power, while low-carbon energy sources are defined as any form of electricity that does not come from thermal power. According to the law of one price, international oil price fluctuations immediately influence Chinese oil prices in both Scenarios 1 and 2. The short and sensitive path that Chinese oil prices take to reach the consumer market, where international oil price primarily influences transportation and communication costs. Oil is also a key component of many chemical products, and is the first link in the industrial production chain, hence, it has a higher influence on the cost of production for the whole chain as well as the price of completed goods. Producers and consumers will often adopt less expensive alternative energy sources in the long term due to relative price fluctuations and substitution effects of various energy sources. Additionally, as economic globalization progresses, fluctuations in international oil prices will indirectly influence variations in energy use through exchange rates. Total oil consumption and international oil prices fluctuate in the opposite direction as a result of these variables, whereas other energy consumption changes in the same way as global oil prices. The price disadvantage of new energy will be amplified if the international oil price falls further and quickly, turning the development of new energy into a challenging or even backward-looking problem. The Chinese government could at this time implement a carbon tax based on energy prices determined by the market to counteract the disadvantage of new energy.

A carbon tax would make consuming fossil fuels more expensive and have the effect of reducing energy use. In Scenarios 3 and 4, it is clear that the consumption of coal and oil and natural gas both dramatically decline following the implementation of a carbon price, but to a lesser amount. Consumption of thermal power and clean electricity both rise, with thermal power’s replacement impact being the most prominent. In the long run, the cost of thermal power will quickly grow as a result of the higher carbon tax, and this, along with the rise in the price of thermal energy, will significantly lessen thermal power’s competitive edge when the cost and price of renewable energy both remain the same or slightly fall. The introduction of the carbon pricing policy will ultimately impede the growth of thermal power. The whole cost of utilizing synthetic energy, which includes direct carbon costs and energy prices, is what determines energy consumption. The carbon tax not only accounts for the cost of carbon emissions directly in the cost of production but also indirectly influences changes in energy prices through transmission into the energy sector’s cost of production. To save costs, businesses will develop or use alternative energy sources to replace fossil fuels, which will help China modify its existing energy balance of coal consumption as the major source of energy and oil consumption as a supplement. The effects of a tax on carbon are not evenly distributed. When oil prices go down, the policy of imposing a tax on carbon can cut energy use in a more efficient manner while also offsetting the increase in consumption of fossil fuels that was caused by the rise in oil prices. Therefore, there is a golden opportunity to establish a carbon tax policy when international oil prices are low and continue low.

### 4.3. Impact Analysis of Scenario Combination on Economy

Due to its high crude oil imports and reliance on outside markets, China’s economy is particularly susceptible to changes in the price of oil globally. Table 2 illustrates the implications of several scenarios on macroeconomic variables. Both Scenario 1 and Scenario 2 show that lower international oil prices promote economic growth, whereas higher prices have the opposite impact and stunt progress. This is owing to the fact that China’s economy is becoming increasingly reliant on oil as it enters a period in which development is driven by consumer upgrading, urbanization, and industrialization. The rise in international oil prices not only increases the production costs of enterprises, but also reduces their profits, makes them reduce investment and production, and at the same time reduces the growth rate of the economy. Although low oil prices are beneficial to China’s macroeconomic stability, sustained low prices will discourage the use of new energy sources that are more costly to develop and encourage an increase in oil consumption, which will lead to an increase in greenhouse gas emissions and other industrial pollutants and slow the green transformation. International oil prices move positively with inflation indicators, such as the CPI and PPI, and there are two main pathways for their impact. First, oil is a direct cost element for both CPI and PPI, meaning that changes in international oil prices will immediately influence the cost of China’s crude oil chemical products, which in turn will affect the cost of operating energy facilities in the country. If oil prices rise, it will drive up the cost of manufacturing and living for everyone. Industries that rely heavily on cheap energy would be hit hardest. Second, fluctuations in oil prices have an effect on the money supply via the flexible exchange rate system and the import/export trade structure, necessitating periodic adjustments to the configuration of both industries and markets. If this keeps happening, inflationary expectations will rise, which will eventually manifest as higher prices.

When the price of oil goes up or down, so does the total social investment. From the standpoint of cost transmission, changes in oil prices will have an immediate effect on the cost of oil for public transportation, machinery, and construction vehicles, which in turn will affect the cost of output in industry and manufacturing. Because of the interconnected nature of the oil and gas sectors, shifts in the price of oil will have repercussions for the natural gas sector, which in turn will influence the oil industry’s downstream businesses. As a result, a significant shift in oil prices will have a stronger effect on investment in adjacent industries since it will alter investors’ expectations of oil prices. Resident income, enterprise revenue, and government income all move in the same direction as international oil prices. However, resident income fluctuates the least, while enterprise income fluctuates the most, and the impact is higher when international oil prices decline than when they rise. Residents’ income is affected by the prices of labor, energy, capital, and other factors of production, and there is a substitution relationship between these components. Changes in energy demand brought about by fluctuations in international oil prices will create fluctuations in the relative pricing of energy and other production components. Corporate revenue is affected by fluctuations in the pricing of capital elements and sectoral gains or losses resulting from volatility in international oil prices. Government revenue changes are related to fluctuations in resident income, firm income, and the prices of other categories. The resident savings, company savings, and government savings exhibit the same direction and size of change as the respective income. Government consumption decreases as international oil prices fall and domestic consumption goes in the opposite direction of international oil prices. The majority of the oil used by people goes toward fueling cars. New energy cars have not caught on in China just yet, causing it to be challenging to discover viable alternatives for auto fuel. As a result, people will limit their use of automobiles and, in turn, lower their oil use as a direct result of the rise in oil prices.

The nominal GDP suffers a decline in both Scenario 3 and Scenario 4. This is a result of the fact that nominal GDP takes into account both labor and capital as well as indirect tax receipts. A decrease in capital revenue and no change in labor revenue is the result of the introduction of a carbon tax, which causes an overall increase in the price of output and an overall decrease in the amount of output. Even if indirect tax income is growing, its proportion of nominal GDP is small. As a direct consequence of this, nominal GDP as a whole experiences a decrease. The real GDP, which is calculated by factoring in investment as well as consumption and net exports, is falling. The implementation of a fee on carbon causes a reduction in residential consumption. Government consumption increases, but due to the low proportion of government consumption in total consumption, Overall, there is a decline in consumption. The carbon tax causes an increase in domestic savings in addition to an increase in the savings of both the government and foreign countries. Investment and savings are equivalent. On the other hand, corporate savings, which make up the bulk of total savings, decline. Total social savings, i.e., total social investment, falls. Because of the carbon tax, the price of domestic items rises relative to the price of international products, while the price of foreign products does not change. This results in fewer net exports. The application of a carbon tax increases government revenue and government transfer payments to households, hence increasing the income of inhabitants. Resident consumption is positively affected by the higher income on the one hand and negatively affected by the carbon tax that raises the price of the product, on the other hand, showing an overall decrease. In general, a carbon tax is advantageous since it increases government income and government investment, hence stimulating economic development. The installation of a carbon tax, on the other hand, is damaging to the incentive for private investment, hence retarding economic growth.

The upstream and downstream of the industrial chain in many industries will be impacted by swings in international oil prices as China’s industrial production capacity expands, bringing increased uncertainty to the operation of linked firms. Table 3 depicts the effect of several scenarios on the output of each industry. In Scenario 1, the production of agriculture, heavy industry, light industry, transportation, and service industry all decline, with agriculture and light industry declining the most. The reason for this is that the increase in international oil prices reduces the quantity of oil inputs and increases the amount of labor inputs in order to save money, hence increasing the price of labor and causing labor-intensive sectors to suffer more severe harm. The sole reason why the rise in international oil prices has contributed to the production of the construction industry is that it will increase the cost of energy utilized by businesses. To save expenses, businesses will convert to substituting energy with inputs of production such as capital or labor. The increase in return on capital investment causes businesses to invest more in fixed assets, with the construction industry benefiting the most as a core component of fixed asset investment. In Scenario 2, the drop in international oil prices encourages the majority of industries to profit from lower intermediate input costs and output expansion. In response to falling international oil prices, domestic refined oil prices change in the same direction, transport costs decrease, and transportation industry output rises. The fall in international oil prices causes enterprises to replace a portion of their capital with oil, although the construction industry has a negligible decline as the economy as a whole improves. Comparing Scenario 1 with Scenario 2, it is evident that the effects of rising and dropping international oil prices on the industrial sector are opposite and asymmetric, and for the majority of industries, the effects of falling international oil prices are stronger.

In Scenario 3 and Scenario 4, the output of agriculture, light industry, and the service industry increase, while the output of heavy industry, construction, and transportation industries decline by varying degrees, with heavy industry and transportation industries experiencing the greatest declines. This is because, on the one hand, heavy industry and transportation have a high energy demand and a large proportion of energy in their inputs, and the imposition of a carbon tax results in a substantial increase in their production costs and a decrease in their supply. Agriculture, light industry, and service industries have relatively small cost increases, therefore the decline in supply is relatively low, and the relative increase in structural prices is small, resulting in an increase in demand. The combined effect of supply and demand results in varying degrees of output growth for the industry. In order to shift the industry’s energy consumption habits from fossil fuels to renewables, a considerable boost from the carbon tax is required. The industry’s affordability and the chilling effect of a carbon tax on growth must be taken into account at the same time. The competitiveness of businesses and the growth of economies may be hampered by a carbon tax that is too high.

### 4.4. Impact Analysis of Scenario Combination on Environment

**Figure 5:**
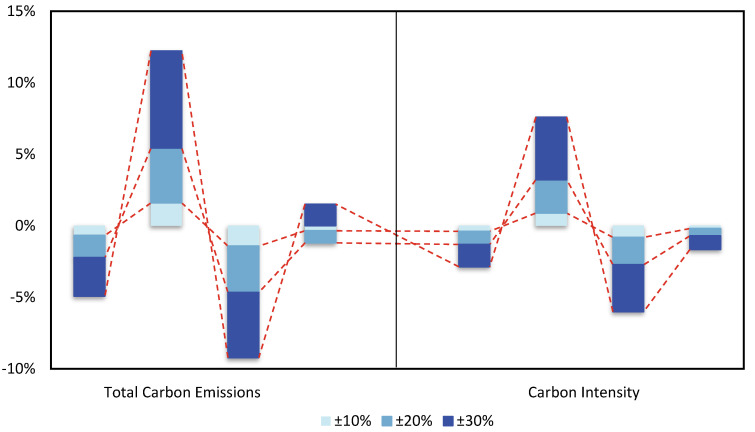
Impact of different scenarios on total carbon emissions and carbon intensity Unit: %.

Figure 5 depicts the implications of several scenarios on total carbon emissions and intensity. In Scenario 1, the increase in the international price of oil directly reduces oil consumption, and businesses explore producing or utilizing cheaper and cleaner energy alternatives, hence lowering carbon emissions. In addition, the increase in international oil prices will have an indirect effect on energy consumption and carbon emissions by influencing macroeconomic and industrial structure. To summarize, the substitution effect between energy variables is what makes high oil costs beneficial to green development. Oil price swings cause shifts in the consumption shares of traditional fossil energy and green energy, and substitution effects occur between energy elements, given a certain level of energy market demand. However, a drop in oil production means consumers will have to pay more to get their hands on the commodity, driving up the cost of living across the board. Because of this, renewable energy sources such as hydropower are gaining popularity among investors, and they are replacing oil in some situations. In Scenario 2, the relationship between fluctuations in international oil prices and changes in the ecological environment demonstrates that a fall in international oil prices will result in a rise in total carbon emissions and carbon emissions intensity. China’s current industrial technology development is skewed toward energy consumption and high energy consumption characteristics, which will inevitably lead to an increase in China’s oil consumption and pollutant emissions in a low oil price environment, which is not conducive to the transition to green growth. However, it cannot be denied that low oil prices enable local customers to obtain cheaper energy items, which is beneficial for expanding strategic oil reserves and decreasing economic expenses. Because of this, it is even more vital to be careful of the oil price increases that follow low oil prices and the test of the possibilities for the growth of renewable energy. To combat the unpredictability of oil prices, it is crucial to implement policies that stimulate the growth of renewable energy sources, such as a carbon tax, before oil prices rise. Abandoning or slowing down green energy investments during periods of low oil prices will increase the cost of future green energy investments, prolong the cycle of green energy development, cause China’s economy to lag behind other nations in terms of green growth, and harm micro-enterprise growth and China’s international competitiveness.

Examining Scenarios 3 and 4, this paper finds that carbon tax policies can successfully reduce the quantity and intensity of carbon emissions. Thus, a carbon tax is an efficient tool for achieving emission reduction goals and limiting fossil energy usage while incentivizing green energy consumption. When oil prices continue to decline, the implementation of a carbon tax policy can effectively offset the rise in carbon emissions caused by the decline in international oil prices. Therefore, China should take advantage of the period of low oil prices to speed up its green growth so that it may actualize ecological civilization and construct a beautiful country. China should hasten the process of energy reform with the aid of a carbon tax policy to advance energy cleanliness and sustainable development during a period of low international oil prices. China has completely launched the market for trading carbon emissions but has not yet implemented a carbon price. Among China’s carbon peak and carbon neutral targets, the goal of carbon neutral in terms of net carbon emissions is definite, as it is to achieve break-even in carbon emissions; however, there is no document that specifies the specific peak target of total carbon dioxide emissions for carbon peak targets. On the basis of the existing carbon market, a carbon tax should be introduced at the appropriate time to form a synergistic combination of policies to, directly and indirectly, regulate carbon emissions, which can effectively reduce the pressure of emission reduction in the carbon market and help achieve the goal of carbon neutrality while promoting the reduction of the peak of total carbon dioxide emissions.

## 5. Conclusions and Policy Recommendations

### 5.1. Conclusions

The mechanism of action of international oil price fluctuations on the energy–economy–environment system is extremely complex due to the substitution and complementarity of different energy sources as well as the differences in the direction of fluctuations of different energy sources influenced by international oil prices. In accordance with the mechanism of action of a combination of policy scenarios affecting energy price volatility elements, such as international oil price swings and greater environmental costs of fossil energy due to the introduction of carbon taxes, the following findings are drawn.

(1) China’s energy imports are substantial and international energy prices are dominated by oil prices. Therefore, an increase in international oil prices leads to an increase in domestic fossil energy prices, which reduces the demand for fossil energy and increases the demand for clean power, resulting in a reduction in overall energy consumption. In contrast, dropping international oil prices have the opposite effect.
(2) A rise in international oil prices increases production costs for firms and produces inflation, resulting in a decrease in local output and investment. A significant decline in international oil prices, on the other hand, results in deflation, which has a negative impact on domestic output and investment. A rise in international oil prices is damaging to labor-intensive businesses, such as light industry and agriculture, whereas a decline in international oil prices will enhance output in industries other than construction.
(3) An increase in international oil prices can mitigate the environmental pollution caused by carbon emissions to some extent, whereas a decrease in international oil prices will result in an increase in the total and intensity of China’s carbon emissions, which is not conducive to China achieving its carbon peak and carbon neutral goals. The influence of international oil price fluctuations in both directions on carbon emissions is not symmetrical.
(4) The carbon tax has a significant impact on reducing fossil energy demand and carbon emissions as well as promoting the demand for clean electricity, and the linkage mechanism between carbon tax and international oil price fluctuations is conducive to enhancing energy saving and emission reduction effects. In other words, when the international price of oil declines dramatically, the carbon tax rate should be increased proportionally, and vice versa.

### 5.2. Policy Recommendations

The following recommendations are offered to China, based on the study’s findings, in order to mitigate the impact of worldwide oil price variations on the energy–economy–environment system from multiple viewpoints.

(1) Encourage the optimization and modernization of energy structure to lessen reliance on oil imports. Reduce the share of high-carbon energy consumption in the energy structure by supporting the optimization and upgrading of the energy structure. Alternately, the market and policy instruments can direct the formation of a reasonable pattern of energy usage.
(2) Implement a multi-directional and multi-channel pattern of oil delivery in order to mitigate the risk of abrupt shifts in international relations and frequent regional conflicts. Additionally, pay particular attention to the trend of oil price fluctuations. When international oil prices decline, it is vital to sustain the rate of oil exploration and development in order to enhance the domestic oil supply capacity and increase oil imports in order to increase strategic oil reserves. When oil prices are high, it is important to sell at the proper time in order to lower the cost of oil imports, boost the pricing power of international oil prices, and mitigate the impact of growing international oil prices on the domestic economy.
(3) When creating legislation about climate change, it is important to consider the rippling impact of oil prices. Continue to reinvent the system of carbon tax regulation and perform a complementary function to carbon emissions trading. In addition to limiting fossil fuel usage, the carbon tax should attempt to increase the efficiency of fossil fuel utilization. The government should assist manufacturers who optimize oil use patterns and create fossil energy technologies in order to further optimize the energy structure, reduce energy intensity, and speed up the greening of the economy.
(4) Against the backdrop of persistently low international oil prices, the government is compelled to employ policy instruments, particularly environmental levies or more direct carbon tax policies. By increasing the price of fossil fuels to lessen the environmental effect caused by their increased consumption, the transition to a low-carbon energy system is facilitated.

## Figures and Tables

**Table 1:** CGE model and I/O table departmental correspondence division.

CGE Model Sector Division	I/0 Table Sector	Sector Number
Agriculture	Agriculture, forestry and fishery	01
Heavy Industry	Metal mining, non-metallic mining and other mineral extraction, chemical industry, non-metallic mineral products, metal smelting and rolling processing industry, metal products, general, special equipment manufacturing, transportation equipment manufacturing, electrical machinery and equipment manufacturing, communications equipment and computer and other electronic equipment manufacturing, instrumentation, other manufacturing products and scrap waste, metalwork machinery and equipment repair	04, 05, 12–23
Light Industry	Food manufacturing and tobacco processing industry, textiles, textiles and clothing, shoes, hats, leather and down and its manufacturing industry, wood processing and furniture manufacturing, paper printing and teaching and sporting goods manufacturing, water production and supply industry	06–10, 26
Architecture	Construction	27
Transportation	Transportation, storage and postal	29
Service industry	Accommodation and catering, information transmission and software and information technology services, finance, real estate, leasing and business services, research and experimental development, integrated technical services, water and environmental and public facilities management, residential services and repair and other services, education, health and social work, culture and sports and recreation, public administration and social security and social organizations	30–42
High-carbon	Coal mining and washing industry, coking industry, petroleum extraction industry, petroleum refining and processing industry, natural gas extraction industry, gas production and supply industry, electricity, heat production and supply industry	02, 03, 11, 24, 25
Low-Carbon	Hydropower, nuclear power, other power supply	24

**Table 2:** Impact of scenario mix on macroeconomic variables Unit: %.

Variables	Scenario 1			Scenario 2			Scenario 3			Scenario 4		
	+10	+20	+30	−10	−20	−30	+10	+20	+30	−10	−20	−30
Nominal GDP	0.75	1.54	2.38	−1.04	−2.39	−2.9	0.61	1.36	2.18	−1.23	−2.64	−3.17
Real GDP	−0.15	−0.23	−0.34	0.21	0.98	1.35	−0.38	−0.52	−0.62	−0.1	0.58	0.91
CPI	0.13	0.19	0.21	−0.19	−0.31	−0.36	0.18	0.26	0.29	−0.11	−0.21	−0.25
PPI	0.64	0.93	1.71	−0.93	−1.52	−2.94	0.75	1.1	1.91	−0.79	−1.29	−2.67
Total Social Savings	1.74	2.93	3.73	−2.6	−3.93	−5.52	1.27	2.33	3.19	−3.24	−4.75	−6.26
Total Social Investment	1.74	2.93	3.73	−2.6	−3.93	−5.52	1.27	2.33	3.19	−3.24	−4.75	−6.26
Government Income	1.02	1.72	2.1	−1.34	−2.03	−2.7	1.47	2.27	2.72	−1.01	−1.63	−2.25
Resident income	0.4	0.53	0.42	−0.53	−0.63	−0.55	0.47	0.61	0.58	−0.48	−0.57	−0.43
Corporate Income	2.56	4.51	5.91	−3.36	−5.32	−7.6	2.06	3.87	5.09	−3.72	−5.79	−8.2
Government Savings	1.02	1.72	2.1	−1.34	−2.03	−2.7	1.47	2.27	2.72	−1.01	−1.63	−2.25
Resident savings	0.4	0.53	0.42	−0.53	−0.63	−0.55	0.47	0.61	0.58	−0.48	−0.57	−0.43
Corporate savings	2.56	4.51	5.91	−3.36	−5.32	−7.6	2.06	3.87	5.09	−3.72	−5.79	−8.2
Government consumption	0.51	0.88	1.09	−0.71	−1.09	−1.28	0.9	1.32	1.65	−0.41	−0.78	−0.89
Resident consumption	−0.17	−0.45	−0.84	0.24	0.56	1.14	−0.21	−0.5	−0.92	0.22	0.52	1.08

**Table 3:** Impact of scenario mix on industry output Unit: %.

Industry	Scenario 1			Scenario 2			Scenario 3			Scenario 4		
	+10	+20	+30	−10	−20	−30	+10	+20	+30	−10	−20	−30
Agriculture	−0.42	−0.76	−1.20	1.04	1.32	2.43	−0.21	−0.45	−0.79	1.28	1.87	3.07
Heavy Industry	−0.30	−0.48	−0.96	0.77	1.46	2.94	−1.12	−1.98	−3.18	−0.25	−0.12	0.73
Light Industry	−0.64	−1.74	−2.54	1.34	1.85	3.10	−0.16	−1.07	−1.59	1.75	2.42	3.77
Architecture	0.47	0.98	1.42	−0.35	−0.63	−1.18	0.16	0.54	0.81	−0.67	−1.16	−2.01
Transportation	−0.36	−1.55	−2.83	0.81	2.02	3.76	−1.03	−2.96	−4.55	0.38	1.12	2.60
Service industry	−0.38	−0.61	−1.15	0.67	1.09	2.02	−0.14	−0.08	−0.08	0.89	1.74	3.01

## Data Availability

Not applicable.
